# Genome Sequences of Three Species of *Hanseniaspora* Isolated from Spontaneous Wine Fermentations

**DOI:** 10.1128/genomeA.01287-16

**Published:** 2016-11-17

**Authors:** Peter R. Sternes, Danna Lee, Dariusz R. Kutyna, Anthony R. Borneman

**Affiliations:** a The Australian Wine Research Institute, Adelaide, South Australia, Australia; b Department of Genetics and Evolution, University of Adelaide, South Australia, Australia

## Abstract

Members of the genus *Hanseniaspora* represent a significant proportion of the normal flora of grape berries and play a significant role in wine fermentation. Here, we present genome sequences for three species of *Hanseniaspora*, *H. opuntiae*, *H. osmophila*, and *H. uvarum*, which were isolated from spontaneous Chardonnay wine fermentation.

## GENOME ANNOUNCEMENT

Members of the genus *Hanseniaspora* represent a significant proportion of the normal flora of grape berries and play a significant role in wine fermentation (1). Besides a role in grape wine fermentation, *Hanseniaspora* species have been used as starter cultures in the fermentation of fruit wines, ciders, and spirits (2–5) and cocoa (6, 7).

One isolate of each of three species of *Hanseniaspora*, AWRI3578 (*H. opuntiae*), AWRI3579 (*H. osmophila*), and AWRI3580 (*H. uvarum*), were obtained from spontaneously fermenting Chardonnay grape must (Adelaide Hills, South Australia, Australia) in 2014. Each isolate was identified to the species level by sequence identity analysis of a fragment of the rDNA internal transcribed spacer (ITS) region (8) against the QIIME UNITE database (ver6_dynamic_s_10.09.2014), with results corroborated against the NCBI nr database using Blast (AWRI3579, 99% identity with *H. osmophila* CBS 313^T^; AWRI3578, 98% identity with *H. opuntiae* CBS 8873^T^; AWRI3580, 100% identity with *H. uvarum* CBS 314^T^).

Sequencing was performed using a combination of Illumina Nextera mate-pair (2- to 5-kb and 6- to 12-kb size selected) and TruSeq PCR-free sequencing libraries that were prepared from purified DNA and run using 2 × 300 bp MiSeq chemistry (Ramaciotti Centre for Functional Genomics, Australia). Sequences for each isolate were assembled using MIRA (version 4.0.2 [http://sourceforge.net/projects/mira-assembler/]) with the resultant contigs (in .ace format) manually refined using SeqManPro (DNAStar, USA).

The genomes of statistics of AWRI3578 (*H. opuntiae*) and AWRI3580 (*H. uvarum*) were very similar (Table 1), while AWRI3579 (*H. osmophila*) produced a far larger but also more fragmented assembly at the contig level; however, the contigs were readily connected by scaffolding to produce a similar number of scaffolds as the other two species.

**TABLE 1:** Genome assembly statistics

Strain	Presumptive species (ITS sequence)	No. of contigs	No. of scaffolds	Assembly size (Mb)	Contig *N*_50_ (kb)	Accession no.
AWRI3578	*Hanseniaspora opuntiae*	67	18	8.83	636	LPNL00000000
AWRI3579	*Hanseniaspora osmophila*	899	17	11.37	8	LPNM00000000
AWRI3580	*Hanseniaspora uvarum*	44	18	8.81	739	LPNN00000000

Augustus annotation (9) predicted 4,176, 4,061, and 4,660 proteins for AWRI3578, AWRI3580, and AWRI3579, respectively. Of these, 3,391, 3,410, and 4,187 proteins could be assigned to OrthoMCL clusters (10, 11). Both the size and predicted protein content of the AWRI3578 and AWWRI3580 genomes are similar to that of *H. valbyensis* (http://genome.jgi.doe.gov/Hanva1_1/Hanva1_1.home.html), while the AWRI3579 genome assembly was similar in size and coding potential to that of *H. vinae* (12). The differences observed in genome size and coding potential are consistent with phylogenies produced by both concatenating 2,045 orthologous proteins predicted in this work from the five species, and from 26S rRNA gene (13), which position *H. opuntiae*, *H. uvarum*, and *H. valbyensis* as a distinct clade and *H. valbyensis* and *H. osmophila* as a separate sister group.

### Accession number(s).

These whole-genome shotgun projects have been deposited in DDBJ/ENA/GenBank under the accession numbers provided in Table 1. The versions described in this paper are the first versions.
